# Exercise Intervention Modulates Synaptic Plasticity by Inhibiting Excessive Microglial Activation *via* Exosomes

**DOI:** 10.3389/fncel.2022.953640

**Published:** 2022-07-19

**Authors:** Chen Li, Jiayi Hu, Wenhong Liu, Changkai Ke, Chuan Huang, Yifan Bai, Bingchen Pan, Junyi Wang, Chunxiao Wan

**Affiliations:** ^1^Department of Physical Medicine and Rehabilitation, Tianjin Medical University General Hospital, Tianjin, China; ^2^School of Basic Medical Sciences, Tianjin Medical University, Tianjin, China; ^3^Tianjin Rehabilitation Center, Tianjin, China; ^4^Department of Rehabilitation Medicine, School of Medicine Technology, Tianjin Medical University, Tianjin, China

**Keywords:** MCAO, exercise intervention, exosomes, synaptic plasticity, microglial

## Abstract

**Background:**

Exosomes can activate microglia to modulate neural activity and synaptic plasticity by phagocytosis of neural spines or synapses. Our previous research found that an early 4-week exercise intervention in middle cerebral artery occlusion (MCAO) rats can promote the release of exosomes and protect the brain. This study intended to further explore the intrinsic mechanism of neuroprotection by exosome release after exercise.

**Methods:**

Rats were randomly divided into four groups: the sham operation (SHAM), middle cerebral artery occlusion (MCAO) with sedentary intervention (SED-MCAO), MCAO with exercise intervention (EX-MCAO), and MCAO with exercise intervention and exosome injection (EX-MCAO-EXO). Modified neurological severity score (mNSS), cerebral infarction volume ratio, microglial activation, dendritic complexity, and expression of synaptophysin (Syn) and postsynaptic density protein 95 (PSD-95) were detected after 28 days of intervention.

**Results:**

(1) The exercise improved body weight and mNSS score, and the survival state of the rats after exosome infusion was better. (2) Compared with the SED-MCAO group, the EX-MCAO (*P* = 0.039) and EX-MCAO-EXO groups (*P* = 0.002) had significantly lower cerebral infarct volume ratios (*P* < 0.05), among which the EX-MCAO-EXO group had the lowest (*P* = 0.031). (3) Compared with the SED-MCAO group, the EX-MCAO and EX-MCAO-EXO groups had a significantly decreased number of microglia (*P* < 0.001) and significantly increased process length/cell (*P* < 0.01) and end point/cell (*P* < 0.01) values, with the EX-MCAO-EXO group having the lowest number of microglia (*P* = 0.036) and most significantly increased end point/cell value (*P* = 0.027). (4) Compared with the SED-MCAO group, the total number of intersections and branches of the apical and basal dendrites in the EX-MCAO and EX-MCAO-EXO groups was increased significantly (*P* < 0.05), and the increase was more significant in the EX-MCAO-EXO group (*P* < 0.05). (5) The expression levels of Syn and PSD-95 in the EX-MCAO (*P*_Syn_ = 0.043, *P*_PSD−95_ = 0.047) and EX-MCAO-EXO groups were significantly higher than those in the SED-MCAO group (*P* < 0.05), and the expression levels in the EX-MCAO-EXO group were significantly higher than those in the EX-MCAO group (*P* < 0.05).

**Conclusion:**

Early exercise intervention after stroke can inhibit the excessive activation of microglia and regulate synaptic plasticity by exosome release.

## Introduction

Stroke is one of the common causes of disability and death worldwide (Yang et al., 2021). Ischemic stroke can lead to interruption of brain energy supply, resulting in nerve cell damage and inflammatory cascade, and finally in neurological dysfunction (del Zoppo, 2009). The ischemic penumbra is activated within a few days of cerebral ischemia (Moskowitz et al., 2010), which is an important time window for cerebral protection. Studies have found that 3 days after ischemia, the brain releases signals of growth factors promoting synaptogenesis, such as growth-associated protein 43 and synaptophysin (Stroemer et al., 1995), which are important for neurological recovery (Coleman et al., 2017).

Exercise is an effective rehabilitation strategy after stroke (Xing and Bai, 2020). After 7 days of grasping training immediately after cerebral ischemia, grasping ability can be restored to the level before injury (Zeiler et al., 2013). The exercise intervention was performed 1 day and 1 week after cerebral ischemia in rats, and it was found that exercise training had the best effect on reducing motor dysfunction after 1 day (Shi et al., 2020).

Exercise promotes exosome release into the circulation (Fruhbeis et al., 2015; Whitham et al., 2018; Brahmer et al., 2019). Both acute (Fruhbeis et al., 2015) and long-term exercises can increase serum exosome levels (Bertoldi et al., 2018; Ma et al., 2018). A study found that exosomes induce long-term brain protection and promote neural recovery (Otero-Ortega et al., 2018).

A previous study by our team confirmed that exercise intervention in the early stage of cerebral infarction in rats (Shi et al., 2020; Shi and Wan, 2021) improved the concentration of exosomes in blood and brain tissues and that the exosomes in blood were targeted to accumulate in the brain, improving neural plasticity and protecting neural function (Li et al., 2021). However, the mechanism by which exosomes enter and function in the brain has not been thoroughly explored.

Studies have found that exosomes can regulate the activation of microglia (Zagrean et al., 2018) by inhibiting the activation of M1 microglia (Li et al., 2019; Zheng et al., 2019; Duan et al., 2020) and regulating neural plasticity. Exosomes derived from microglia can significantly reduce cerebral infarct volume and dysfunction (Song et al., 2019a). Microglia have a key role in regulating synaptic connections and synaptic remodeling (Frost and Schafer, 2016; Yu et al., 2021). Studies have found that after cerebral ischemia, microglia can directly contact synapses for a long time (Wake et al., 2009), directly phagocytose spines and synapses, and induce synaptic formation to regulate neural activity and synaptic plasticity (Crapser et al., 2021; Hanslik et al., 2021; Sancho et al., 2021; Wang and Li, 2021). However, whether the exercise-promoted release of exosomes modulates synaptic plasticity *via* microglia is unclear.

In this study, mNSS score, cerebral infarction volume ratio, microglial activation, and synaptic plasticity changes were detected after 4 weeks of intervention in different groups. In this way, regulation of synaptic plasticity by inhibition of excessive activation of microglia by exosomes was found to be the mechanism of early exercise intervention after stroke.

## Materials and Methods

### Experimental Animals and Groups

Forty-eight adult male Sprague–Dawley rats (8–10 weeks old and weighing 280–320 g; Beijing Huafukang Biotechnology Co., Ltd., China) were used in this study. The experimental procedure was performed according to the National Institutes of Health (NIH) Laboratory Care and Use Guidelines to minimize the number of animals used and pain in the animals during the experiment and was approved by the Laboratory Animal Welfare Ethics Committee of Tianjin Medical University General Hospital (IRB2021-DWFL-403).

**Figure 1:**
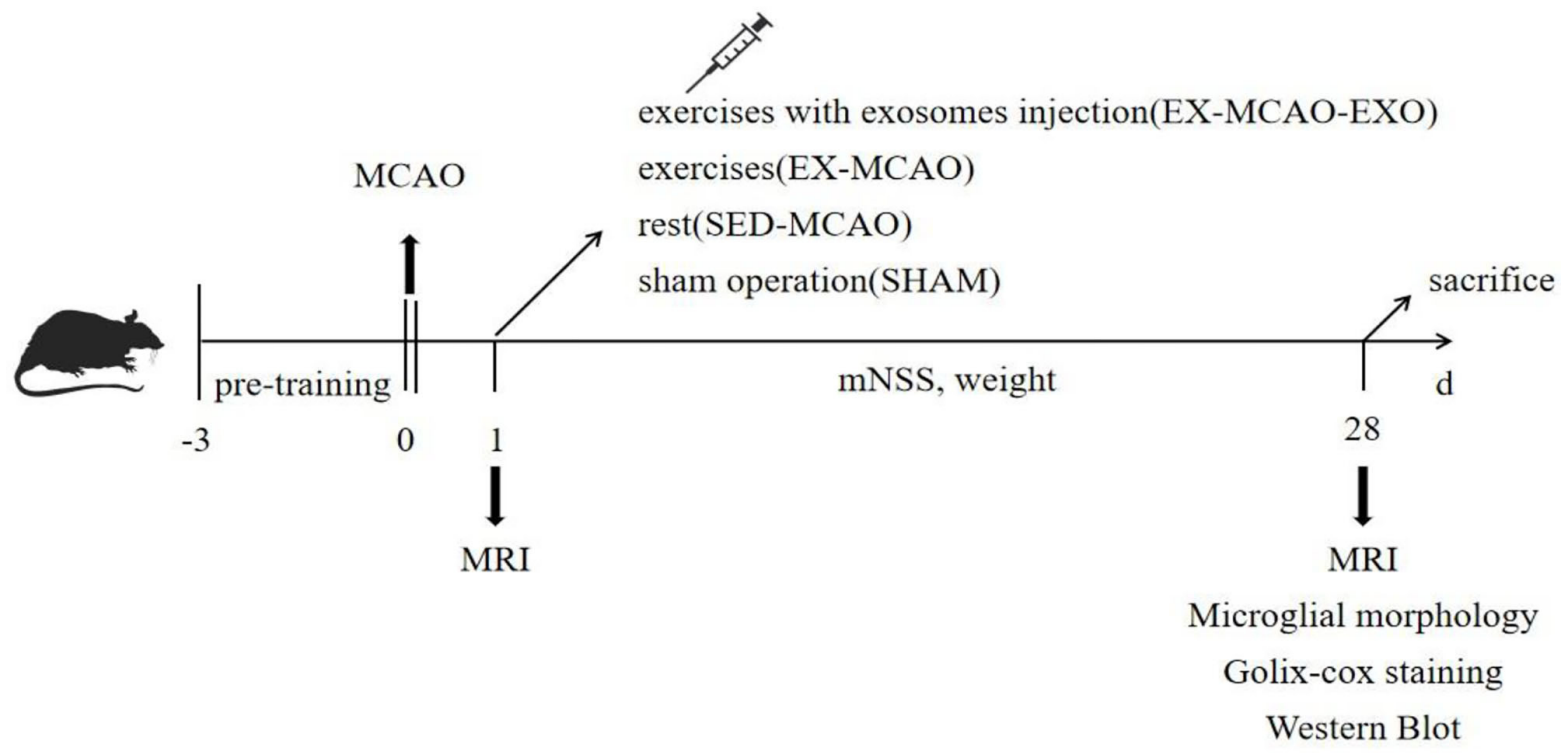
Schematic of the experimental protocol.

The rats were randomly divided into four groups: the sham operation (SHAM), MCAO with sedentary intervention (SED-MCAO), MCAO with exercise intervention (EX-MCAO), and MCAO with exercise intervention and exosome injection (EX-MCAO-EXO). The schematic of the protocol is shown in Figure 1.

### MCAO Model

The modified Longa thread embolization method was used to prepare the MCAO model (Longa et al., 1989). After the rats were anesthetized, the external carotid artery and the proximal end of the common carotid artery were ligated, and a fine silicon-coated surgical nylon monofilament was inserted from the common carotid artery to the internal carotid artery for 60 min (Beijing Xinong Technology Co.; Ltd., 2838-A4, 0.38 ± 0.02 mm). Longa score was determined after 24 h, and those with 1–3 points were included in this experiment.

### Brain-Derived Exosome Extraction

Brain tissues were ground into a homogenate and centrifuged at 1,500 × g for 20 min and 13,000 × g for 3 min to thoroughly remove cellular debris. Next, supernatants were filtered through a.22-μm-pore-size filter to exclude particles > 220 nm in diameter. Ultracentrifugation was performed for 2 h at 100,000 × *g*, and precipitates were resuspended in PBS (Ngolab et al., 2017). All centrifugation steps were performed at 4°C.

### Exercise Intervention

The exercise program adopts the previous research program of the team (Shi et al., 2020; Li et al., 2021). All the rats were randomly divided into groups after 3 days of treadmill preintervention. The exercise intervention started 1 day after MCAO. The rats were made to exercise on a treadmill (ZS-PT of Zhongshi Dichuang Company, Beijing, China, angle of 0°, speed of 12 m/min) for 30 min each time, 5 times/week, for a total of 4 weeks.

Rats in the EX-MCAO-EXO group received brain-derived exosomes containing 100 μg of protein through tail vein injection 1 d after MCAO (Xin et al., 2013; Zhang et al., 2015; Safakheil and Safakheil, 2020; Li et al., 2021) and then underwent the same exercise intervention as the EX-MCAO group.

### Neurological Function Assessment and Weight

Changes in body weight and neurological function of rats in each group were detected 1, 3, 7, 14, 21, and 28 d after MCAO. The modified neurological severity score (mNSS) was used for neurological scoring and ranged from 0 to 18. The mNSS test is a classic method for comprehensively assessing sensory, motor, balance, and reflexes in MCAO rats. The more severe the neurological deficit in rats, the higher the score.

### MRI Scan

Before the exercise intervention and after 28 days of intervention, the rats were anesthetized (3% isoflurane, nasal inhalation anesthesia). Magnetic resonance scanning (9.4T, Bruker BioSpec94/30 UER+PET insert, Germany) was conducted to collect T2-weighted images (T2WI, SE sequence, FOV = 35 × 35 mm, matrix = 256 × 256 mm, TR = 2,500 ms, TE = 33 ms, thickness = 0.8 mm, and slices = 20). Infarct volume ratio = (total volume of contralateral – uninfarcted volume of ipsilateral)/total volume of contralateral.

### Microglial Immunofluorescence Staining

Brain tissue was soaked in 4% paraformaldehyde for 12 h and then removed, placed in 15 and 30% sucrose solutions for complete dehydration, rinsed with PBS, and blotted dry. Brain tissue sections were prepared at a thickness of 10 μm on a cryostat (Leica CM1860, Germany) at −22 °C. The frozen sections were rewarmed for 20 min, washed thrice in PBS, ruptured with 0.3% Triton X-100 (Solarbio, China) for 30 min, and blocked with 3% Albumin Bovine V (Solarbio, China) for 1 h. Then, the sections were incubated with the primary antibody against Iba1 (1:500; Abcam, United Kingdom) diluted with Antibody Diluent (Solarbio, China) at 4°C overnight. A goat anti-rabbit Alexa Fluor 488 secondary antibody (1:200; Invitrogen, United States) was added to the sections, and they were incubated for 1 h at room temperature. DAPI (Abcam, United Kingdom) was added dropwise for staining and mounting. Images of the peri-infarct area were taken using an inverted fluorescence microscope (Olympus IX73, Japan). Microglia were skeletonized using the method of Young and Morrison; that is, immunofluorescence images were background-removed, binarized, and skeletonized using the ImageJ software. Then, the skeletonized microglial morphology was analyzed (Young and Morrison, 2018).

### Golgi-Cox Staining

Golgi-Cox staining (FD Rapid Golgi Staining Kit; FD Neuro Technologies, United States) was performed to observe changes in dendrites in the infarcted penumbra cortex (Hu et al., 2020). Dendritic structures were analyzed by laser confocal scanning microscopy (Olympus FV1000, Japan). The Fiji software (https://imagej.net/Fiji) was used for neuron tracking. Sholl analysis was conducted to analyze trajectory, automatically drawing a concentric circle with the cell body as the center, with a step of 10 μm. The complexity of dendrites was quantified by the number of intersections and the number of neuron branches.

### Western Blot Analysis

Total protein was extracted from peri-infarcted brain tissues, and total protein concentration was determined with a BCA kit (Solarbio, China). Proteins were separated by polyacrylamide gel electrophoresis (SDS–PAGE) and blotted on polyvinylidene fluoride (PVDF) membranes. The membranes were incubated with 5% non-fat milk for 1 h at room temperature and with anti-Syn (1:2,000; Abcam, United Kingdom), PSD-95 (1:1,000; Affinity, China), and β-tubulin (1:3,000; Solarbio, China) antibodies at 4°C overnight. Then, the cells were incubated with the IgG antibody (1:1,000; Cell Signaling Technology, Danvers, MA, United States) for 1 h at room temperature. Visualization was performed on a gel electrophoresis imager using an ECL hypersensitive chemiluminescent solution (Millipore, Germany).

### Statistical Analysis

Statistical software SPSS 25.0 (SPSS Inc., Armonk, NY, United States) and GraphPad Prism 6.0 (GraphPad Software, Inc., La Jolla, CA, United States) were used. Data are presented as means ± standard deviations (SD). The Shapiro–Wilks test was performed to verify the normal distribution of the data. A one-way analysis of variance (ANOVA) was carried out to compare multiple groups, and an LSD *t*-test was performed for multiple comparisons between pairs. *P* < 0.05 was considered significant.

## Results

### Body Weight and MNSS Score of Rats

The body weight and neurological score of each group were detected 1, 3, 7, 14, 21, and 28 d after the MCAO.

**Figure 2:**
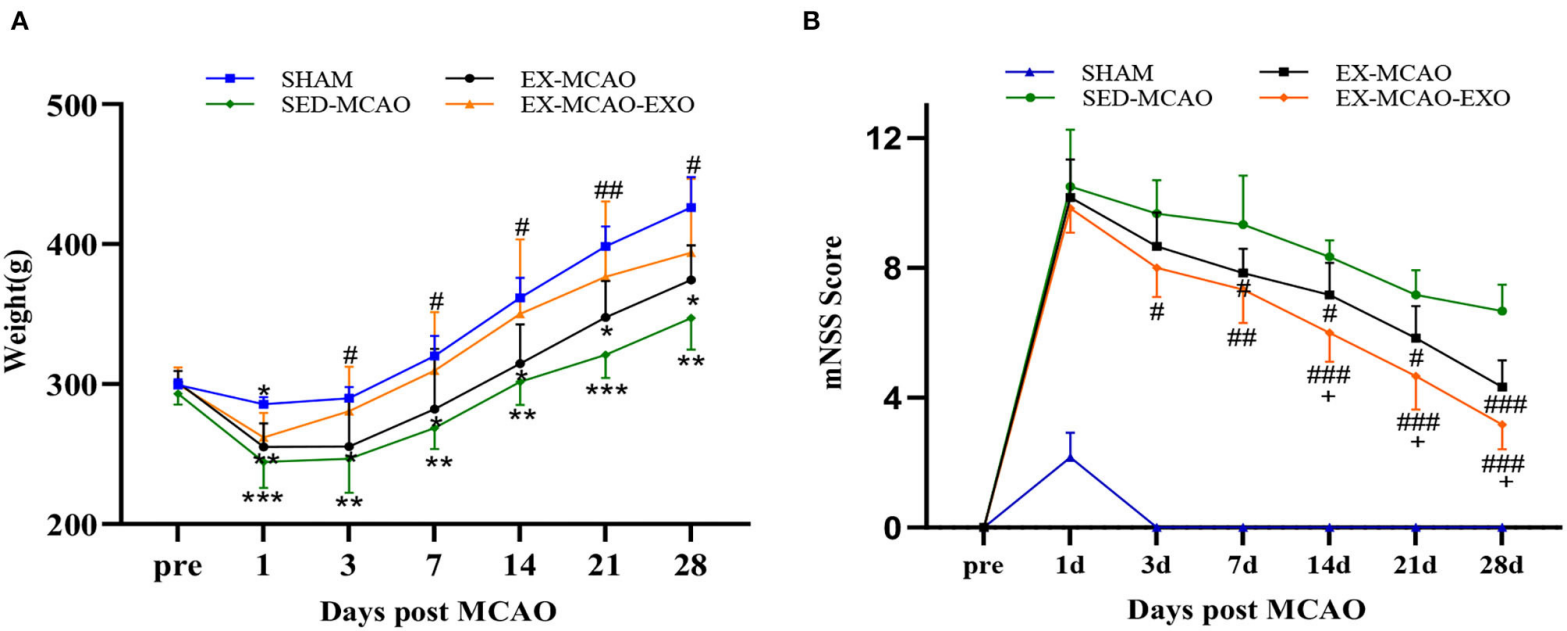
Survival status and mNSS scores of the MCAO rats. **(A)** Weight changes in the rats. **(B)** Changes in behavioral scores of the rats. *P < 0.05, **P < 0.01, and ***P < 0.001 compared with the SHAM group; ^#^*P* < 0.05, ^##^*P* < 0.01, and ^###^*P* < 0.001 compared with the SED-MCAO group; ^+^*P* < 0.05 compared with the EX-MCAO group. Mean ± standard deviation. N = 6/group.

In the comparison of body weight (Figure 2A), the body weight of rats in each group increased with time throughout the experiment, and the body weights of all of the MCAO groups were lower than that of the SHAM group (*P* < 0.05). Compared with those in the SED-MCAO group, the body weights in the EX-MCAO and EX-MCAO-EXO groups increased, and the body weight in the EX-MCAO-EXO group increased significantly on the 3rd day (280.5 ± 12.97 vs. 246.67 ± 9.91, *P* < 0.05).

The comparison of mNSS scores (Figure 2B) showed that compared with that of the SED-MCAO group, the mNSS of the EX-MCAO group decreased significantly on day 7 (7.83 ± 0.31 vs. 9.33 ± 0.62, *P* < 0.05) and the mNSS of the EX-MCAO-EXO group decreased significantly on day 3 (8 ± 0.37 vs. 9.67 ± 0.42, *P* < 0.05). The score of the EX-MCAO-EXO group was significantly lower than that of the EX-MCAO group on day 14 (6 ± 0.37 vs. 7.17 ± 0.4, *P* < 0.05).

### Cerebral Infarction Volume Ratio

**Figure 3:**
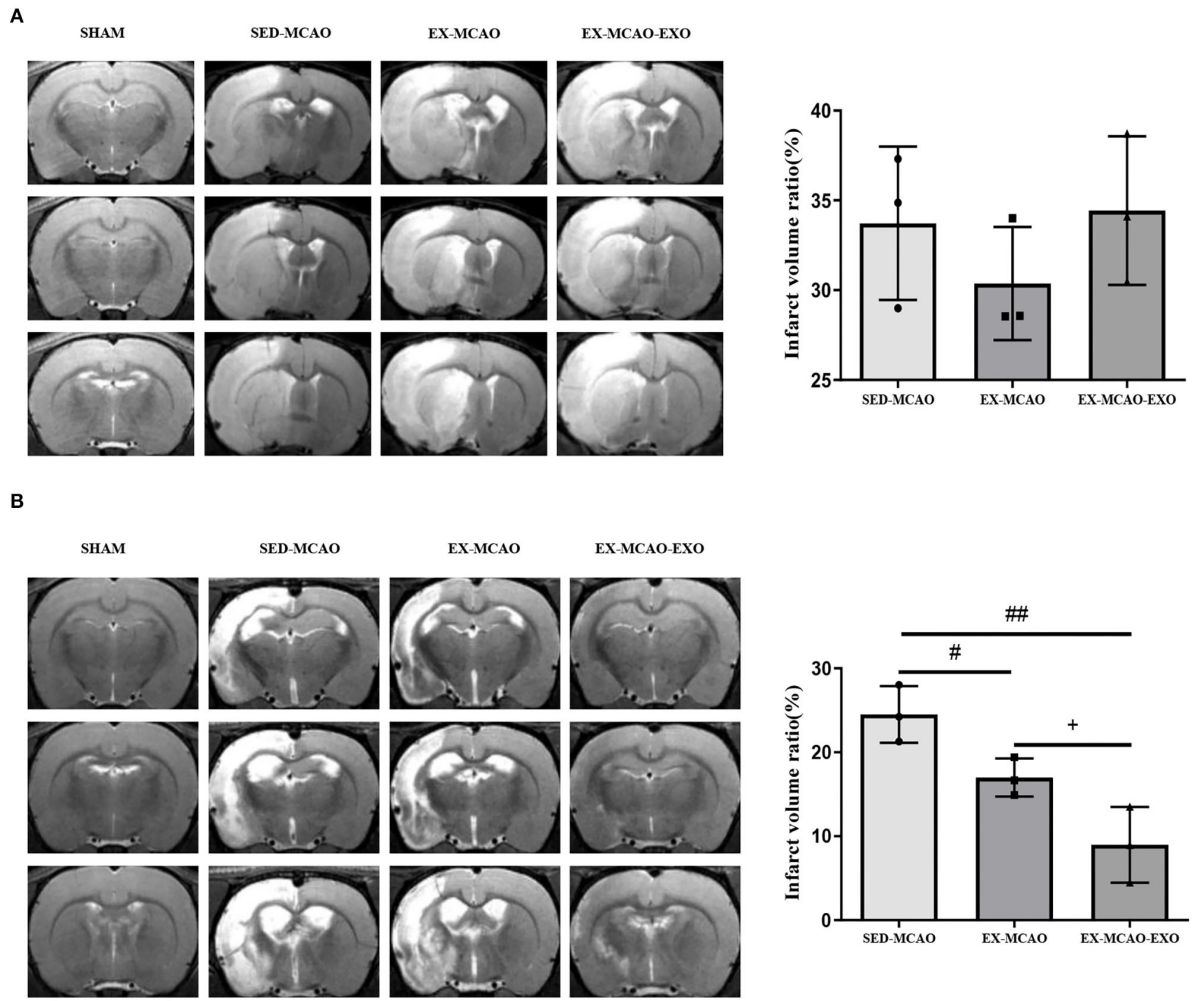
Cerebral infarct volume ratio. **(A)** T2WI images of the rats and the cerebral infarct volume ratio in each group before intervention. **(B)** T2-WI images of the rats and the cerebral infarct volume ratio in each group after 28 days of intervention. ^#^*P* < 0.05 and ^##^*P* < 0.01 compared with the SED-MCAO group; ^+^*P* < 0.05 compared with the EX-MCAO group. Mean ± standard deviation. *N* = 3/group.

There was no significant difference in the cerebral infarction volume ratio between the experimental groups before the intervention (*P* > 0.05, Figure 3A). After 28 days of the intervention (Figure 3B), compared with the SED-MCAO group, the EX-MCAO (16.99 ± 1.31 vs. 24.52 ± 1.95%, *P* < 0.05) and EX-MCAO-EXO groups had a significantly decreased cerebral infarction volume ratio (8.98 ± 2.6 vs. 24.52 ± 1.95%, *P* < 0.01). Compared with the EX-MCAO group, the cerebral infarct volume ratio of the EX-MCAO-EXO group was significantly decreased (*P* = 0.031).

### Microglia Activation

**Figure 4:**
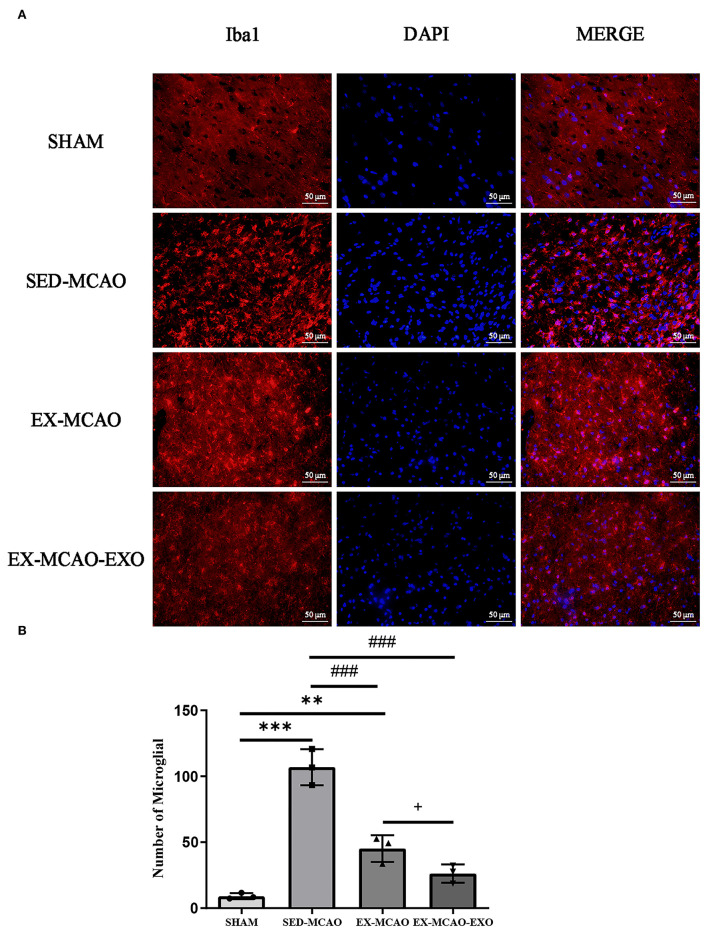
**(A)** Representative immunofluorescence images of Iba1 staining. Bar = 50 μm. **(B)** Number of microglia. **P < 0.01 and ***P < 0.001 compared with the SHAM group; ^###^*P* < 0.001 compared with the SED-MCAO group; ^+^*P* < 0.05 compared with the EX-MCAO group. Mean ± standard deviation. *N* = 3/group.

After 28 days of the intervention (Figure 4), the results of fluorescent staining of microglia in the peri-infarcted area of the brain showed that the number of microglia in the SED-MCAO (106.89 ± 7.89 vs. 9.11 ± 1.31, *P* < 0.001) and EX-MCAO (45.22 ± 5.86 vs. 9.11 ± 1.31, *P* < 0.01) groups was increased significantly when compared with the SHAM group, and there was no significant change in the EX-MCAO-EXO group (26.22 ± 4.01 vs. 9.11 ± 1.31, P=0.053). Compared with the SED-MCAO group, the number of microglia in the EX-MCAO and EX-MCAO-EXO groups was significantly decreased (*P* < 0.001), and the EX-MCAO-EXO group had the lowest number (*P* = 0.036).

**Figure 5:**
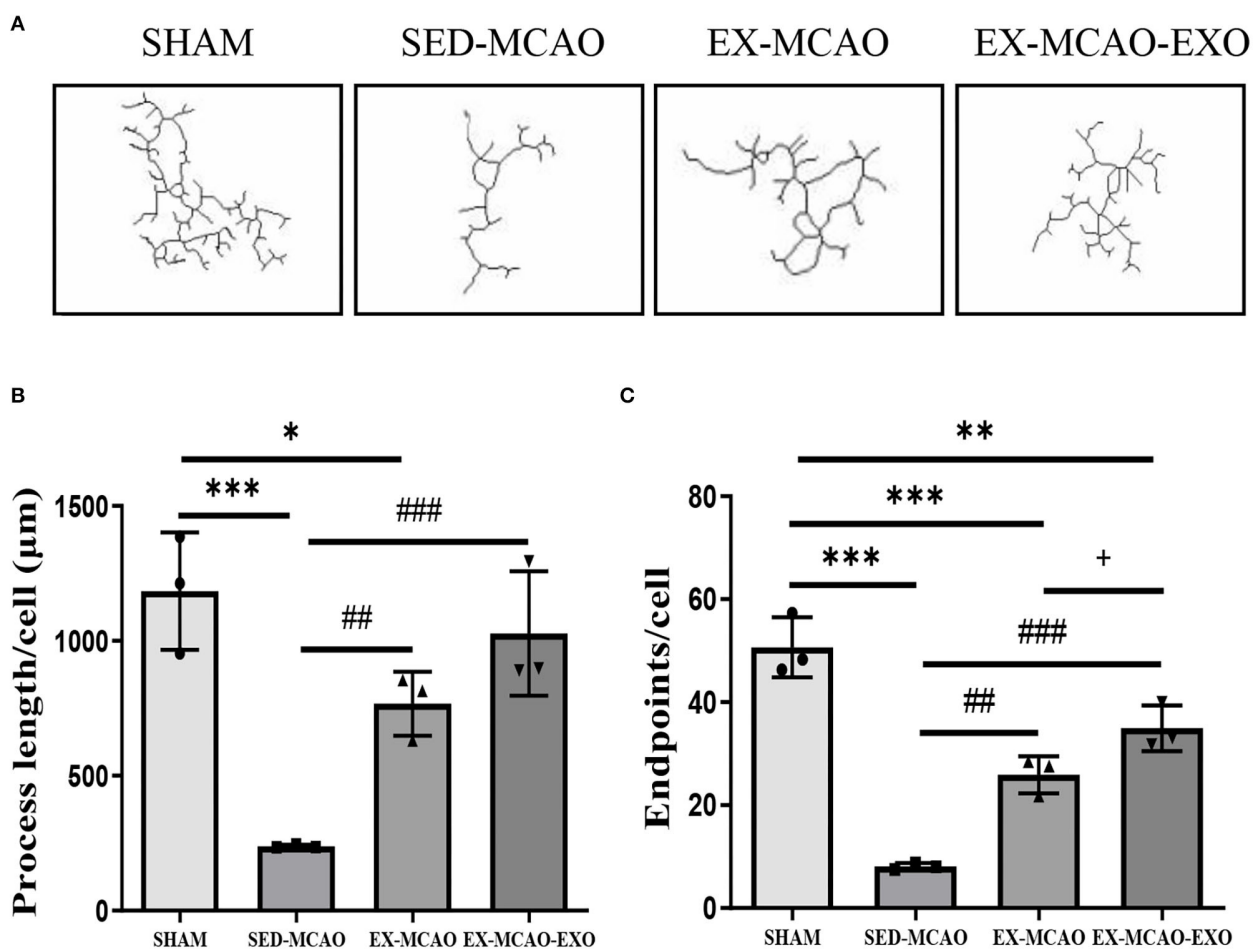
Microglial morphological analysis. **(A)** Representative image of microglial skeletonization. **(B)** Process length/cell. **(C)** End point/cell. *P < 0.05, **P < 0.01, and ***P < 0.001 compared with the SHAM group; ^##^*P* < 0.01 and ^###^*P* < 0.001 compared with the SED-MCAO group; ^+^*P* < 0.05 compared with the EX-MCAO group. Mean ± standard deviation. *N* = 3/group.

The morphological characteristics of microglia in the peri-infarcted area of the brain after 28 days of the intervention are shown in Figure 5A.

The process length/cell assessment is shown in Figure 5B. Compared with the SHAM group, the process length/cell in the SED-MCAO (238.64 ± 3.64 vs. 1,183.91 ± 125.63, *P* < 0.001) and EX-MCAO (766.49 ± 68.61 vs. 1,183.91 ± 125.63, *P* < 0.05) groups was significantly decreased, and the EX-MCAO-EXO group had no significant difference (1,027.46 ± 133.36 vs. 1,183.91 ± 125.63, *P* = 0.291). Process length/cell was significantly higher in the EX-MCAO and EX-MCAO-EXO groups than in the SED-MCAO group (*P* < 0.01). There was no significant difference between the EX-MCAO-EXO and EX-MCAO groups (*P* = 0.096).

The endpoints/cell assessment is shown in Figure 5C. The end point/cell in the MCAO groups was lower than that in the SHAM group (*P* < 0.01). Compared with the SED-MCAO group, end point/cell was significantly increased in the EX-MCAO (25.91 ± 2.08 vs. 8.11 ± 0.37, *P* < 0.01) and the EX-MCAO-EXO groups (34.95 ± 2.55 vs. 8.11 ± 0.37, *P* < 0.001), among which the EX-MCAO-EXO group had the highest end point/cell (*P* = 0.027).

### Neuronal Dendrite Characteristics

**Figure 6:**
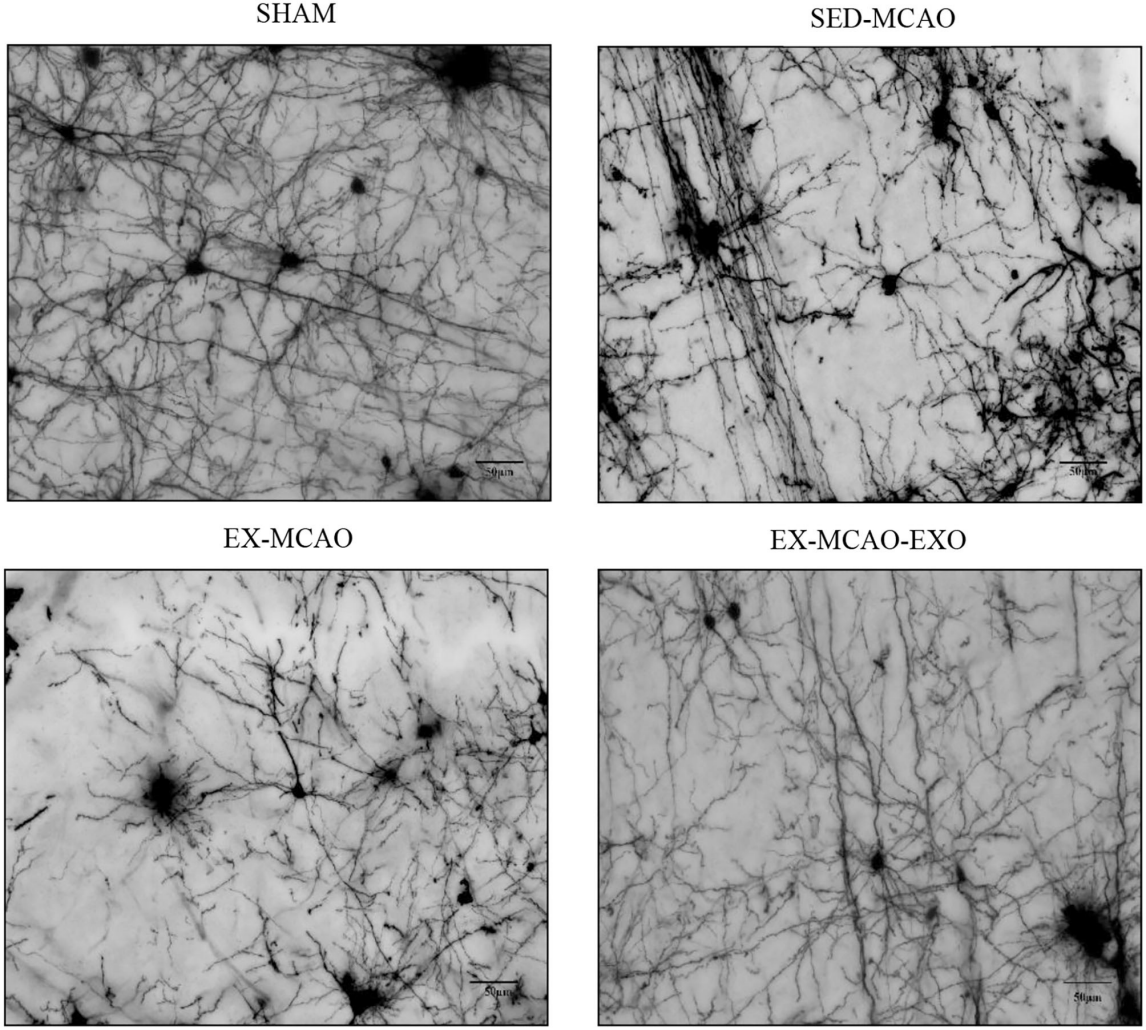
Representative images of neuronal dendrites in the peri-infarct cortex. Bar = 50 μm.

**Figure 7:**
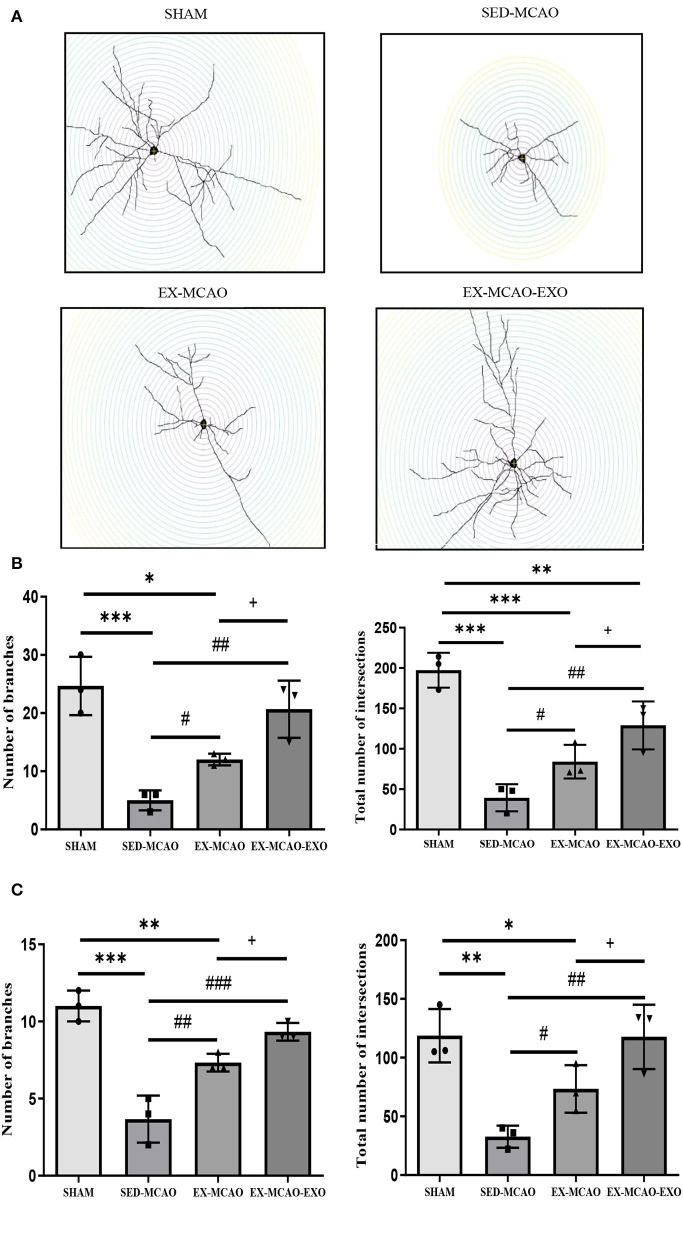
Characteristics of basal and apical dendrites in each group were analyzed by Sholl analysis. **(A)** Representative images of each group for Sholl analysis, with a step of 10 μm. **(B)** Total number of branches and intersections in basal dendrites. **(C)** Total number of branches and intersections in apical dendrites. *P < 0.05, **P < 0.01, and ***P < 0.001 compared with the SHAM group; ^#^*P* < 0.05, ^##^*P* < 0.01, and ^###^*P* < 0.001 compared with the SED-MCAO group; ^+^*P* < 0.05 compared with the EX-MCAO group. Mean ± standard deviation. *N* = 3/group.

Representative images of neuronal dendrites in the peri-infarct cortex were obtained using by using Golgi staining (Figure 6). Sholl analysis of the images (Figure 7A) revealed the number of branches and intersections between apical and basal dendrites (Table 1).

**Table 1:** Characteristics of basal and apical dendrites in each group.

		**SHAM**	**SED-MCAO**	**EX-MCAO**	**EX-MCAO-EXO**
Basal dendrites	Number of branches	24.67 ± 2.91	5.00 ± 9.68	12.00 ± 0.58	20.67 ± 2.85
	Total number of intersections	197.33 ± 12.44	39.33 ± 9.68	84.00 ± 12.01	129.00 ± 17.16
Apical dendrites	Number of branches	11.00 ± 0.58	3.67 ± 0.88	7.33 ± 0.33	9.33 ± 0.33
	Total number of intersections	118.67 ± 13.17	32.67 ± 5.46	73.33 ± 11.67	117.67 ± 15.84

Further statistics on these parameters found that whether in basal or apical dendrites, the number of branches and intersections in the SED-MCAO and EX-MCAO groups was significantly lower than that in the SHAM group (*P* < 0.05). Compared with the SED-MCAO group, the number of branches and intersections in the EX-MCAO and EX-MCAO-EXO groups was significantly increased (*P* < 0.05), and the increase was more significant in the EX-MCAO-EXO group (*P* < 0.05).

### The Expression of Synaptic Plasticity Proteins

**Figure 8:**
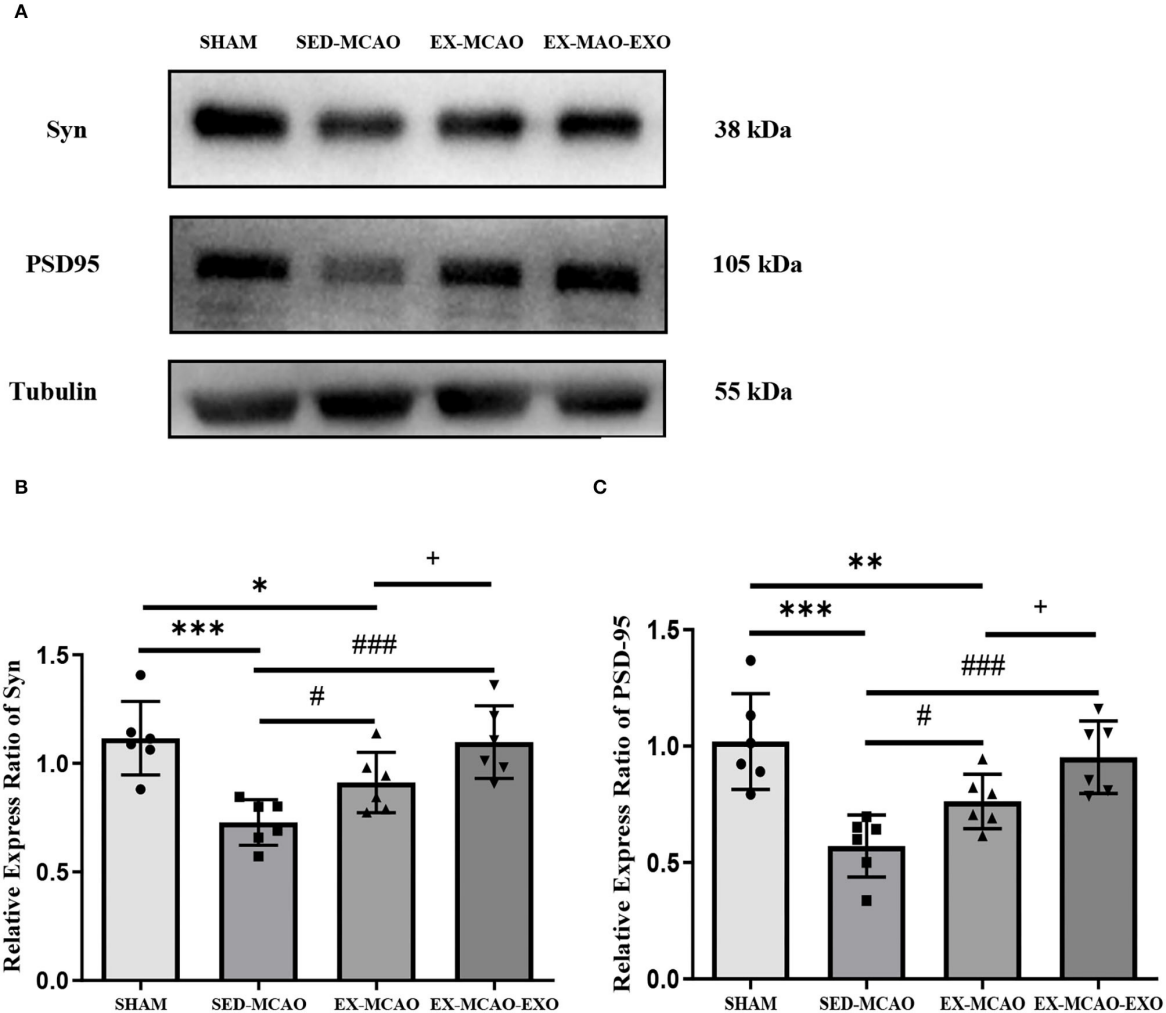
Synaptic plasticity protein expression. **(A)** Western blot. **(B)** Syn relative expression level. **(C)** PSD-95 relative expression level. *P < 0.05, **P < 0.01, and ***P < 0.001 compared with the SHAM group; ^#^*P* < 0.05 and ^###^*P* < 0.001 compared with the SED-MCAO group; ^+^*P* < 0.05 compared with the EX-MCAO group. Mean ± standard deviation. *N* = 6/group.

After 28 days of the intervention (Figure 8), the protein expression levels of Syn and PSD-95 in the SED-MCAO (Syn: 0.73 ± 0.03 vs. 1.12 ± 0.07, *P* < 0.001; PSD-95: 0.57 ± 0.05 vs. 1.02 ±0.08, *P* < 0.001) and EX-MCAO (Syn: 0.91 ± 0.06 vs. 1.12 ± 0.07, *P* < 0.05; PSD-95: 0.76 ± 0.05 vs. 1.02 ± 0.08, *P* < 0.01) groups were significantly lower than that in the SHAM group, but there was no significant difference between the EX-MCAO-EXO and the SHAM groups (Syn: 1.1 ± 0.07 vs. 1.12 ± 0.07, *P* > 0.05; PSD-95: 0.95 ± 0.06 vs. 1.02 ± 0.08, *P* > 0.05). The protein expression levels of Syn and PSD-95 in the EX-MCAO and EX-MCAO-EXO groups were significantly higher than those in the SED-MCAO group (*P* < 0.05), and the expression level in the EX-MCAO-EXO group was the highest (*P* < 0.05).

## Discussion

This study found that after 28 days of exercise intervention in MCAO rats: (1) the rats gained weight and had improved neurological function, (2) the cerebral infarct volume ratio decreased, (3) excessive microglial activation was significantly inhibited, (4) the total number of dendritic intersections and branches in the peripheral area of the infarction was significantly improved, and (5) the protein expression levels of Syn and PSD-95 in the peripheral area of infarction were increased. Compared with the simple exercise group, the exosome infusion combined with the exercise group showed a further improvement in the above indicators.

### Exercise Intervention Improves Body Weight and Neurological Function in MCAO Rats, and Exosomes Are Involved in the Process of Neural Recovery

Stroke often results in neurological deficits and weight loss, negatively affecting prognosis and function (Yang et al., 2019). Numerous studies have shown that exercising early (days to weeks) after stroke improves neurological function in rats (Coleman et al., 2017). Our previous study found that an exercise intervention started 1 day after stroke has the best improvement effect (Shi et al., 2020); exercise can promote the release of circulating exosomes that enter ischemic brain tissues and promote brain tissue recruitment of more exosomes (Li et al., 2021). In this study, compared with the SED-MCAO group, mNSS was decreased significantly in the EX-MCAO group from the 7th day and decreased significantly in the EX-MCAO-EXO group from the 3rd day, and mNSS in the EX-MCAO-EXO group was significantly lower than that in the EX-MCAO group from the 14th day. Therefore, we speculate that exosomes play a role in improving neurological function and participate in the process of neural repair caused by exercise.

### Exercise Intervention Improves the Cerebral Infarction Volume Ratio and Exosomes Participate in the Process of Brain Structure Repair

Before the intervention, there was no significant difference in the ratio of cerebral infarct volume among the groups. The exercise intervention can reduce the volume of cerebral infarction. Two weeks of moderate-intensity treadmill exercise can significantly reduce the volume of cerebral infarction (Pan et al., 2021). After 24 h of ischemia–reperfusion, both mild and severe exercises can significantly reduce the volume of cerebral infarction (Li et al., 2020). Cheng et al. found that treadmill exercise promotes neurogenesis, enhances myelin recovery, and reduces cerebral infarct volume (Cheng et al., 2020). Zheng et al. showed that exosomes can significantly reduce the infarction volume ratios of ischemic rats 6 h after MCAO (Zheng et al., 2019) and have neuroprotective effects (Zhou et al., 2020). Our study found that infarct volume could be reduced 28 days after the intervention and that infarct volume was further reduced after exosome infusion, which further indicated the special role of exosomes in an exercise intervention.

### Exercise Intervention Inhibits the Excessive Activation of Microglia, and Exosomes Are Involved in the Inhibitory Process

Microglia play a dual role in ischemic brain injury (Ran et al., 2021). On the one hand, after cerebral ischemia, microglia respond rapidly to the microenvironment of the brain (Dong et al., 2021), change from a resting state (“branching” with pruning synaptic function) to an activated state (“amoeba” with phagocytic function), and migrate to the ischemic region to protect neurons by forming phagocytic fragments (Shi and Pamer, 2011). On the other hand, excessive activation of microglia releases inflammatory factors and cytotoxic substances that exacerbate brain damage (Ma et al., 2017; Xing and Bai, 2020).

Exercise inhibits microglial activation (Liu et al., 2022). Liu C et al. found that activation of microglia was inhibited after swimming training (Liu et al., 2021a). Kodali et al. found that intermittent running could reverse the excessive activation of microglia and increase their branches (Kodali et al., 2021). This study found that after MCAO, the number of microglia significantly increased, and process length/cell and end point/cell values decreased, suggesting that microglia were activated and transformed into “amoeba.” After exercise intervention, microglial activation was inhibited, and microglial activation was further inhibited by exosome input.

Studies have found that exosomes can inhibit microglial activation. Moss L et al. found that intranasal exosomes inhibit microglial activation (Moss et al., 2021). Plasma exosomes inhibit LPS-induced microglial inflammatory response (Wang et al., 2021). Bone marrow mesenchymal stem cell-derived exosomes alleviate neuroinflammation by modulating microglial activation (Liu et al., 2021b).

A previous study by our team confirmed that exercise can promote the release and migration of exosomes into the brain (Li et al., 2021), so we speculate that exosomes are involved in the process of exercise intervention inhibiting the activation of microglia.

### Inhibition of Microglial Activation Promotes Synaptic Remodeling, and Exosomes Regulate Synaptic Plasticity

Resting microglia can participate in the formation and pruning of neuronal synapses (Peferoen et al., 2014). Crapser J et al. suggested that microglia may prune synapses by engulfing the extracellular matrix surrounding the synapses (Crapser et al., 2021). Microglia have been found to mediate changes in synaptic plasticity (Innes et al., 2019) through the processes of neurogenesis and axogenesis (Yu et al., 2021). Activation of M2 microglia enhances neuroplasticity and angiogenesis and promotes functional recovery (Song et al., 2019b). Inhibition of M1 microglial activation enhances synaptic plasticity (Sun et al., 2020). Leech et al. found that inhibition of excessive microglial activation increases dendritic spine density (Leech et al., 2020).

Improving synaptic connections between neurons is particularly important for recovery after ischemic brain injury (Dabrowski et al., 2019). Approximately 90% of excitatory synapses are located on dendrites, and the morphological structure and complexity of dendrites affect the connection between synapses and the efficiency of signal transmission (Nguyen et al., 2021). This study found that dendritic complexity was reduced and that the expression levels of Syn and PSD-95 were reduced after MCAO, suggesting that synaptic structure was disrupted and synaptic function was compromised. The exercise intervention increased the expression of dendritic complexity and synaptic plasticity-related proteins, and synaptic plasticity was further enhanced after exosome input.

Exosomes can regulate synaptic plasticity through microglia. Studies have shown that exosomes can regulate neuroplasticity (Xin et al., 2013) and neuronal development and maintain myelin and synaptic function in peripheral brain infarcts by enhancing neurite remodeling (Domingues et al., 2020), thus affecting the morphology and function of microglia (Vogel et al., 2018; Garcia et al., 2022) and the density of dendritic spines (Sobue et al., 2018). Combined with our previous studies, we speculate that exercise can regulate synaptic plasticity by inhibiting the excessive activation of microglia after exosomes enter the brain.

## Limitations of the Study

More time points were not tested to observe the dynamic changes in the experimental indicators. We have not performed related experiments with exosome inhibitors, and this will be further explored in future studies.

## Summary

Early exercise intervention after stroke can inhibit the excessive activation of microglia by exosomes, thereby regulating synaptic plasticity and protecting neural function.

## Data Availability Statement

The raw data supporting the conclusions of this article will be made available by the authors, without undue reservation.

## Ethics Statement

The animal study was reviewed and approved by Laboratory Animal Welfare Ethics Committee of Tianjin Medical University General Hospital (IRB2021-DWFL-403).

## Author Contributions

CL, JH, WL, CK, CH, YB, BP, and JW performed the experiments. CL wrote the manuscript. All authors listed have made a substantial, direct, and intellectual contribution to the work and approved it for publication.

## Funding

This study was supported by the Natural Science Foundation of Tianjin, China (Grant No: 18JCZDJC98900), Tianjin Key Medical Discipline (Specialty) Construction Project, and General Project of Tianjin Natural Science Foundation Multi-Investment Fund Project.

## Conflict of Interest

The authors declare that the research was conducted in the absence of any commercial or financial relationships that could be construed as a potential conflict of interest.

## Publisher's Note

All claims expressed in this article are solely those of the authors and do not necessarily represent those of their affiliated organizations, or those of the publisher, the editors and the reviewers. Any product that may be evaluated in this article, or claim that may be made by its manufacturer, is not guaranteed or endorsed by the publisher.

## References

[B1] Bertoldi K. Cechinel L. R. Schallenberger B. Corssac G. B. Davies S. Guerreiro I. C. K.. (2018). Circulating extracellular vesicles in the aging process: impact of aerobic exercise. Mol. Cell Biochem. 440, 115–125. 10.1007/s11010-017-3160-4 28819811

[B2] Brahmer A. Neuberger E. Esch-Heisser L. Haller N. Jorgensen M. M. Baek R.. (2019). Platelets, endothelial cells and leukocytes contribute to the exercise-triggered release of extracellular vesicles into the circulation. J. Extracell. Vesicl. 8, 1615820. 10.1080/20013078.2019.1615820 31191831 PMC6542154

[B3] Cheng J. Shen W. Jin L. Pan J. Zhou Y. Pan G.. (2020). Treadmill exercise promotes neurogenesis and myelin repair via upregulating Wnt/betacatenin signaling pathways in the juvenile brain following focal cerebral ischemia/reperfusion. Int. J. Mol. Med. 45, 1447–1463. 10.3892/ijmm.2020.4515 32323740 PMC7138282

[B4] Coleman E. R. Moudgal R. Lang K. Hyacinth H. I. Awosika O. O. Kissela B. M.. (2017). Early rehabilitation after stroke: a narrative review. Curr. Atheroscler. Rep. 19, 59. 10.1007/s11883-017-0686-6 29116473 PMC5802378

[B5] Crapser J. D. Arreola M. A. Tsourmas K. I. Green K. N. (2021). Microglia as hackers of the matrix: sculpting synapses and the extracellular space. Cell Mol. Immunol. 18, 2472–2488. 10.1038/s41423-021-00751-3 34413489 PMC8546068

[B6] Dabrowski J. Czajka A. Zielinska-Turek J. Jaroszynski J. Furtak-Niczyporuk M. Mela A.. (2019). Brain functional reserve in the context of neuroplasticity after stroke. Neural. Plast. 2019, 9708905. 10.1155/2019/9708905 30936915 PMC6415310

[B7] del Zoppo G. J. (2009). Inflammation and the neurovascular unit in the setting of focal cerebral ischemia. Neuroscience. 158, 972–982. 10.1016/j.neuroscience.2008.08.028 18824084 PMC2665879

[B8] Domingues H. S. Falcao A. M. Mendes-Pinto I. Salgado A. J. Teixeira F. G. (2020). Exosome circuitry during (De)(Re)myelination of the central nervous system. Front. Cell Dev. Biol. 8, 483. 10.3389/fcell.2020.00483 32612996 PMC7308472

[B9] Dong R. Huang R. Wang J. Liu H. Xu Z. (2021). Effects of microglial activation and polarization on brain injury after stroke. Front. Neurol. 12, 620948. 10.3389/fneur.2021.620948 34276530 PMC8280287

[B10] Duan S. Wang F. Cao J. Wang C. (2020). Exosomes derived from MicroRNA-146a-5p-enriched bone marrow mesenchymal stem cells alleviate intracerebral hemorrhage by inhibiting neuronal apoptosis and microglial M1 polarization. Drug. Des. Devel. Ther. 14, 3143–3158. 10.2147/DDDT.S255828 32821084 PMC7425091

[B11] Frost J. L. Schafer D. P. (2016). Microglia: architects of the developing nervous system. Trends Cell Biol. 26, 587–597. 10.1016/j.tcb.2016.02.006 27004698 PMC4961529

[B12] Fruhbeis C. Helmig S. Tug S. Simon P. Kramer-Albers E. M. (2015). Physical exercise induces rapid release of small extracellular vesicles into the circulation. J. Extracell. Vesicles. 4, 28239. 10.3402/jev.v4.28239 26142461 PMC4491306

[B13] Garcia G. Fernandes A. Stein F. Brites D. (2022). Protective signature of ifngamma-stimulated microglia relies on miR-124-3p regulation from the secretome released by mutant APP swedish neuronal cells. Front. Pharmacol. 13, 833066. 10.3389/fphar.2022.833066 35620289 PMC9127204

[B14] Hanslik K. L. Marino K. M. Ulland T. K. (2021). Modulation of glial function in health, aging, and neurodegenerative disease. Front. Cell Neurosci. 15, 718324. 10.3389/fncel.2021.718324 34531726 PMC8439422

[B15] Hu J. Li C. Hua Y. Liu P. Gao B. Wang Y.. (2020). Constraint-induced movement therapy improves functional recovery after ischemic stroke and its impacts on synaptic plasticity in sensorimotor cortex and hippocampus. Brain Res. Bull. 160, 8–23. 10.1016/j.brainresbull.2020.04.006 32298779

[B16] Innes S. Pariante C. M. Borsini A. (2019). Microglial-driven changes in synaptic plasticity: A possible role in major depressive disorder. Psychoneuroendocrinology. 102, 236–247. 10.1016/j.psyneuen.2018.12.233 30594100

[B17] Kodali M. Mishra V. Hattiangady B. Attaluri S. Gonzalez J. J. Shuai B.. (2021). Moderate, intermittent voluntary exercise in a model of Gulf War Illness improves cognitive and mood function with alleviation of activated microglia and astrocytes, and enhanced neurogenesis in the hippocampus. Brain Behav. Immun. 97, 135–149. 10.1016/j.bbi.2021.07.005 34245811 PMC9885810

[B18] Leech T. Apaijai N. Palee S. Higgins L. A. Maneechote C. Chattipakorn N.. (2020). Acute administration of metformin prior to cardiac ischemia/reperfusion injury protects brain injury. Eur. J. Pharmacol. 885, 173418. 10.1016/j.ejphar.2020.173418 32750367

[B19] Li C. Ke C. Su Y. Wan C. (2021). Exercise intervention promotes the growth of synapses and regulates neuroplasticity in rats with ischemic stroke through exosomes. Front. Neurol. 12, 752595. 10.3389/fneur.2021.752595 34777222 PMC8581302

[B20] Li F. Geng X. Huber C. Stone C. Ding Y. (2020). In search of a dose: the functional and molecular effects of exercise on post-stroke rehabilitation in rats. Front. Cell Neurosci. 14, 186. 10.3389/fncel.2020.00186 32670026 PMC7330054

[B21] Li Z. Liu F. He X. Yang X. Shan F. Feng J.. (2019). Exosomes derived from mesenchymal stem cells attenuate inflammation and demyelination of the central nervous system in EAE rats by regulating the polarization of microglia. Int. Immunopharmacol. 67, 268–280. 10.1016/j.intimp.2018.12.001 30572251

[B22] Liu C. P. Zhong M. Sun J. X. He J. Gao Y. Qin F. X.. (2021a). miR146a reduces depressive behavior by inhibiting microglial activation. Mol. Med. Rep. 23, 1–11. 10.3892/mmr.2021.12102 33880591 PMC8097766

[B23] Liu M. X. Luo L. Fu J. H. He J. Y. Chen M. Y. He Z. J.. (2022). Exercise-induced neuroprotection against cerebral ischemia/reperfusion injury is mediated via alleviating inflammasome-induced pyroptosis. Exp. Neurol. 349, 113952. 10.1016/j.expneurol.2021.113952 34921847

[B24] Liu X. Zhang M. Liu H. Zhu R. He H. Zhou Y.. (2021b). Bone marrow mesenchymal stem cell-derived exosomes attenuate cerebral ischemia-reperfusion injury-induced neuroinflammation and pyroptosis by modulating microglia M1/M2 phenotypes. Exp. Neurol. 341, 113700. 10.1016/j.expneurol.2021.113700 33741350

[B25] Longa E. Z. Weinstein P. R. Carlson S. Cummins R. (1989). Reversible middle cerebral artery occlusion without craniectomy in rats. Stroke. 20, 84–91. 10.1161/01.STR.20.1.84 2643202

[B26] Ma C. Wang J. Liu H. Chen Y. Ma X. Chen S.. (2018). Moderate exercise enhances endothelial progenitor cell exosomes release and function. Med. Sci. Sports Exerc. 50, 2024–2032. 10.1249/MSS.0000000000001672 30222687

[B27] Ma Y. Wang J. Wang Y. Yang G. Y. (2017). The biphasic function of microglia in ischemic stroke. Prog. Neurobiol. 157, 247–272. 10.1016/j.pneurobio.2016.01.005 26851161

[B28] Moskowitz M. A. Lo E. H. Iadecola C. (2010). The science of stroke: mechanisms in search of treatments. Neuron. 67, 181–198. 10.1016/j.neuron.2010.07.002 20670828 PMC2957363

[B29] Moss L. D. Sode D. Patel R. Lui A. Hudson C. Patel N. A.. (2021). Intranasal delivery of exosomes from human adipose derived stem cells at forty-eight hours post injury reduces motor and cognitive impairments following traumatic brain injury. Neurochem. Int. 150, 105173. 10.1016/j.neuint.2021.105173 34453976 PMC8511339

[B30] Ngolab J. Trinh I. Rockenstein E. Mante M. Florio J. Trejo M.. (2017). Brain-derived exosomes from dementia with Lewy bodies propagate alpha-synuclein pathology. Acta Neuropathol. Commun. 5, 46. 10.1186/s40478-017-0445-5 28599681 PMC5466770

[B31] Nguyen L. D. Fischer T. T. Ehrlich B. E. (2021). Pharmacological rescue of cognitive function in a mouse model of chemobrain. Mol. Neurodegener. 16, 41. 10.1186/s13024-021-00463-2 34174909 PMC8235868

[B32] Otero-Ortega L. Gomez de Frutos M. C. Laso-Garcia F. Rodriguez-Frutos B. Medina-Gutierrez E. Lopez J. A.. (2018). Exosomes promote restoration after an experimental animal model of intracerebral hemorrhage. J. Cereb. Blood Flow Metab. 38, 767–779. 10.1177/0271678X17708917 28524762 PMC5987932

[B33] Pan G. Cheng J. Shen W. Lin Y. Zhu A. Jin L.. (2021). Intensive treadmill training promotes cognitive recovery after cerebral ischemia-reperfusion in juvenile rats. Behav. Brain Res. 401, 113085. 10.1016/j.bbr.2020.113085 33358915

[B34] Peferoen L. Kipp M. van der Valk P. van Noort J. M. Amor S. (2014). Oligodendrocyte-microglia cross-talk in the central nervous system. Immunology. 141, 302–313. 10.1111/imm.12163 23981039 PMC3930369

[B35] Ran Y. Su W. Gao F. Ding Z. Yang S. Ye L.. (2021). Curcumin ameliorates white matter injury after ischemic stroke by inhibiting microglia/macrophage pyroptosis through nf-kappab suppression and NLRP3 inflammasome inhibition. Oxid. Med. Cell Longev. 2021, 1552127. 10.1155/2021/1552127 34630845 PMC8497115

[B36] Safakheil M. Safakheil H. (2020). The effect of exosomes derived from bone marrow stem cells in combination with rosuvastatin on functional recovery and neuroprotection in rats after ischemic stroke. J. Mol. Neurosci. 70, 724–737. 10.1007/s12031-020-01483-1 31974756

[B37] Sancho L. Contreras M. Allen N. J. (2021). Glia as sculptors of synaptic plasticity. Neurosci. Res. 167, 17–29. 10.1016/j.neures.2020.11.005 33316304 PMC8513541

[B38] Shi C. Pamer E. G. (2011). Monocyte recruitment during infection and inflammation. Nat. Rev. Immunol. 11, 762–774. 10.1038/nri3070 21984070 PMC3947780

[B39] Shi Y. Huang C. Su Y. Jiang J. Y. Wan C. X. (2020). Effect of early exercise intervention on corticospinal tract in rats with cerebral infarction. Chin. J. Phys. Med. Rehabil. 42, 583–587. 10.3760/cma.j.issn.0254-1424.2020.07.002

[B40] Shi Y. Wan C. (2021). Compensation of ipsilateral motor and sensory functions by contralateral uncrossed pathway in a stroke patient with half brain. Am. J. Phys. Med. Rehabil. 100, e4–e8. 10.1097/PHM.0000000000001432 33534220

[B41] Sobue A. Ito N. Nagai T. Shan W. Hada K. Nakajima A.. (2018). Astroglial major histocompatibility complex class I following immune activation leads to behavioral and neuropathological changes. Glia. 66, 1034–1052. 10.1002/glia.23299 29380419

[B42] Song D. Zhang X. Chen J. Liu X. Xue J. Zhang L.. (2019b). Wnt canonical pathway activator TWS119 drives microglial anti-inflammatory activation and facilitates neurological recovery following experimental stroke. J. Neuroinflammation. 16, 256. 10.1186/s12974-019-1660-8 31810470 PMC6896312

[B43] Song Y. Li Z. He T. Qu M. Jiang L. Li W.. (2019a). M2 microglia-derived exosomes protect the mouse brain from ischemia-reperfusion injury via exosomal miR-124. Theranostics. 9, 2910–2923. 10.7150/thno.30879 31244932 PMC6568171

[B44] Stroemer R. P. Kent T. A. Hulsebosch C. E. (1995). Neocortical neural sprouting, synaptogenesis, and behavioral recovery after neocortical infarction in rats. Stroke. 26, 2135–2144. 10.1161/01.STR.26.11.2135 7482662

[B45] Sun H. He X. Tao X. Hou T. Chen M. He M.. (2020). The CD200/CD200R signaling pathway contributes to spontaneous functional recovery by enhancing synaptic plasticity after stroke. J. Neuroinflammation. 17, 171. 10.1186/s12974-020-01845-x 32473633 PMC7260848

[B46] Vogel A. Upadhya R. Shetty A. K. (2018). Neural stem cell derived extracellular vesicles: Attributes and prospects for treating neurodegenerative disorders. EBioMedicine. 38, 273–282. 10.1016/j.ebiom.2018.11.026 30472088 PMC6306394

[B47] Wake H. Moorhouse A. J. Jinno S. Kohsaka S. Nabekura J. (2009). Resting microglia directly monitor the functional state of synapses in vivo and determine the fate of ischemic terminals. J. Neurosci. 29, 3974–3980. 10.1523/JNEUROSCI.4363-08.2009 19339593 PMC6665392

[B48] Wang X. L. Li L. (2021). Microglia regulate neuronal circuits in homeostatic and high-fat diet-induced inflammatory conditions. Front. Cell Neurosci. 15, 722028. 10.3389/fncel.2021.722028 34720877 PMC8549960

[B49] Wang Y. Gao C. Gao T. Zhao L. Zhu S. Guo L.. (2021). Plasma exosomes from depression ameliorate inflammation-induced depressive-like behaviors via sigma-1 receptor delivery. Brain Behav. Immun. 94, 225–234. 10.1016/j.bbi.2021.02.004 33607235

[B50] Whitham M. Parker B. L. Friedrichsen M. Hingst J. R. Hjorth M. Hughes W. E.. (2018). Extracellular vesicles provide a means for tissue crosstalk during exercise. Cell Metab. 27, 237–51. e4. 10.1016/j.cmet.2017.12.001 29320704

[B51] Xin H. Li Y. Cui Y. Yang J. J. Zhang Z. G. Chopp M.. (2013). Systemic administration of exosomes released from mesenchymal stromal cells promote functional recovery and neurovascular plasticity after stroke in rats. J. Cereb. Blood Flow Metab. 33, 1711–1715. 10.1038/jcbfm.2013.152 23963371 PMC3824189

[B52] Xing Y. Bai Y. A. (2020). Review of exercise-induced neuroplasticity in ischemic stroke: pathology and mechanisms. Mol. Neurobiol. 57, 4218–4231. 10.1007/s12035-020-02021-1 32691303

[B53] Yang J. Kim E. Beltran C. Cho S. (2019). Corticosterone-mediated body weight loss is an important catabolic process for poststroke immunity and survival. Stroke. 50, 2539–2546. 10.1161/STROKEAHA.119.026053 31345131 PMC6710102

[B54] Yang L. Qian J. Yang B. He Q. Wang J. Weng Q.. (2021). Challenges and improvements of novel therapies for ischemic stroke. Front. Pharmacol. 12, 721156. 10.3389/fphar.2021.721156 34658860 PMC8514732

[B55] Young K. Morrison H. (2018). Quantifying microglia morphology from photomicrographs of immunohistochemistry prepared tissue using imagej. J. Vis. Exp. 5, e57648. 10.3791/57648 29939190 PMC6103256

[B56] Yu F. Huang T. Ran Y. Li D. Ye L. Tian G.. (2021). New insights into the roles of microglial regulation in brain plasticity-dependent stroke recovery. Front. Cell Neurosci. 15, 727899. 10.3389/fncel.2021.727899 34421544 PMC8374071

[B57] Zagrean A. M. Hermann D. M. Opris I. Zagrean L. Popa-Wagner A. (2018). Multicellular crosstalk between exosomes and the neurovascular unit after cerebral ischemia. therapeutic implications. Front. Neurosci. 12, 811. 10.3389/fnins.2018.00811 30459547 PMC6232510

[B58] Zeiler S. R. Gibson E. M. Hoesch R. E. Li M. Y. Worley P. F. O'Brien R. J.. (2013). Medial premotor cortex shows a reduction in inhibitory markers and mediates recovery in a mouse model of focal stroke. Stroke. 44, 483–489. 10.1161/STROKEAHA.112.676940 23321442 PMC4086919

[B59] Zhang Y. Chopp M. Meng Y. Katakowski M. Xin H. Mahmood A.. (2015). Effect of exosomes derived from multipluripotent mesenchymal stromal cells on functional recovery and neurovascular plasticity in rats after traumatic brain injury. J. Neurosurg. 122, 856–867. 10.3171/2014.11.JNS14770 25594326 PMC4382456

[B60] Zheng Y. He R. Wang P. Shi Y. Zhao L. Liang J.. (2019). Exosomes from LPS-stimulated macrophages induce neuroprotection and functional improvement after ischemic stroke by modulating microglial polarization. Biomater. Sci. 7, 2037–2049. 10.1039/C8BM01449C 30843911

[B61] Zhou S. Gao B. Sun C. Bai Y. Cheng D. Zhang Y.. (2020). Vascular endothelial cell-derived exosomes protect neural stem cells against ischemia/reperfusion injury. Neuroscience. 441, 184–196. 10.1016/j.neuroscience.2020.05.046 32502570

